# Prevalence, Diversity, and Load of *Borrelia* species in Ticks That Have Fed on Humans in Regions of Sweden and Åland Islands, Finland with Different Lyme Borreliosis Incidences

**DOI:** 10.1371/journal.pone.0081433

**Published:** 2013-11-21

**Authors:** Peter Wilhelmsson, Pontus Lindblom, Linda Fryland, Jan Ernerudh, Pia Forsberg, Per-Eric Lindgren

**Affiliations:** 1 Division of Medical Microbiology, Department of Clinical and Experimental Medicine, Faculty of Health Sciences, Linköping University, Linköping, Sweden; 2 Division of Clinical Immunology, Department of Clinical and Experimental Medicine, Faculty of Health Sciences, Linköping University, Linköping, Sweden; 3 Division of Infectious Diseases, Department of Clinical and Experimental Medicine, Faculty of Health Sciences, Linköping University, Linköping, Sweden; 4 Clinic of Infectious Diseases, County Council of Östergötland, Linköping, Sweden; 5 Medical Services, Department of Microbiology, Ryhov County Hospital, Jönköping, Sweden; Kansas State University, United States of America

## Abstract

The incidence of Lyme borreliosis (LB) in a region may reflect the prevalence of *Borrelia* in the tick population. Our aim was to investigate if regions with different LB incidences can be distinguished by studying the prevalence and diversity of *Borrelia* species in their respective tick populations. The *Borrelia* load in a feeding tick increases with the duration of feeding, which may facilitate a transmission of *Borrelia* Spirochetes from tick to host. Therefore, we also wanted to investigate how the *Borrelia* load in ticks that have fed on humans varies with the duration of tick feeding. During 2008 and 2009, ticks that had bitten humans were collected from four regions of Sweden and Finland, regions with expected differences in LB incidence. The duration of tick feeding was estimated and *Borrelia* were detected and quantified by a quantitative PCR assay followed by species determination. Out of the 2,154 *Ixodes ricinus* ticks analyzed, 26% were infected with *Borrelia* and seven species were identified. *B. spielmanii* was detected for the first time in the regions. The tick populations collected from the four regions exhibited only minor differences in both prevalence and diversity of *Borrelia* species, indicating that these variables alone cannot explain the regions’ different LB incidences. The number of *Borrelia* cells in the infected ticks ranged from fewer than ten to more than a million. We also found a lower number of *Borrelia* cells in adult female ticks that had fed for more than 36 hours, compared to the number of *Borrelia* cells found in adult female ticks that had fed for less than 36 hours.

## Introduction

Ticks transmit a wide range of infections to humans, including Lyme borreliosis (LB) and relapsing fever borreliosis (RF). LB is a multi-systemic inflammatory disease caused by spirochetes belonging to the *Borrelia burgdorferi* sensu lato (sl) complex, which comprises at least 18 species [1]. In Europe, 8 species of this complex have been reported: *B. afzelii*, *B. garinii*, *B. burgdorferi* sensu stricto (ss), *B. valaisiana, B. lusitaniae*, *B. spielmanii, B. bavariensis* and *B. bissettii*. Among them, *B. afzelii*, *B. garinii*, and *B. burgdorferi* ss are the most frequently reported in human clinical specimens. *Borrelia burgdorferi* sl are predominantly transmitted by *Ixodes* ticks. *Borrelia miyamotoi*, a member of the relapsing fever group has been detected in *Ixodes ricinus* ticks [2] and has recently been associated with disease in humans [3,4], characterized by recurring episodes of fever. *Ixodes ricinus*, the predominant *Ixodes* species in Western Europe, has three active life stages and may feed on more than 200 animal species, including mammals, birds, and reptiles [5]. Different groups of animals are more or less suitable as hosts to certain life stages of the *I. ricinus* [6,7], and more or less suitable as reservoirs to certain *Borrelia* species [8,9]. Each life stage of the tick may therefore harbor a certain diversity of *Borrelia* species.

Two meta-analysis studies demonstrate that countries with high LB incidences (e.g. Austria, Czech Republic, Slovenia, Switzerland, and Sweden) also have a high prevalence of *Borrelia* in “their” field-collected tick populations, while countries with low LB incidences (e.g. France, Great Britain, Italy, and Poland) have a low prevalence of *Borrelia* in “their” field-collected tick populations [10,11]. Even if ticks collected from field are considered to be host-seeking it is not evident that they would attach to and bite a human that passes by. Besides, the methods used to collect ticks in field are influenced by weather conditions both before and during sampling [12]. Further, the different life stages of *I. ricinus* have different seasonal host-seeking patterns that are quite variable and not yet fully understood [13,14]. From a clinical point of view it would be more relevant to study the prevalence and diversity of *Borrelia* in ticks that have actually bitten humans, and only few such European studies exist [2,15–17]. It would also be interesting to evaluate if such prevalence reflects the incidences of LB in the same area as the tick bite occurred. To investigate this, a direct comparison of the *Borrelia* content in ticks from different regions with dissimilar incidences of LB is required.

The duration of tick feeding influences the risk to get infected with *Borrelia* after a tick bite. Crippa et al. (2008) observed that *B. burgdorferi* ss was only transferred to mice when *Ixodes* nymphs fed for more than 48 hours [18]. Further, one out of seven and four out of eight mice exposed to *B. afzelii* infected ticks for 24 hours and 48 hours, respectively, became infected; thereafter the transmission risk increased. The duration of tick feeding also influences the *Borrelia* load in a feeding tick. Piesman et al. (2001) observed a sixfold increase of *B. burgdorferi* ss cells when *Ixodes* nymphs had fed on mice for 48 hours [19]. All together, this means that a longer duration of tick feeding leads to a higher load of *Borrelia* in the tick that could facilitate a transmission of *Borrelia* spirochetes from tick to host; the higher load the higher infection probability. To our knowledge, the *Borrelia* load in ticks that have bitten humans has never been related to the duration of tick feeding. Such investigation will improve our understanding of when a feeding tick may be able to transmit *Borrelia* spirochetes to humans. 

An ongoing prospective Nordic epidemiological investigation, denoted the Tick-Borne Diseases (TBD) STING-study, was initiated in 2007 [2,20]. The overall aim of the TBD STING-study is to investigate the prevalence of tick-borne pathogens in ticks that have fed on humans and to evaluate if parameters such as pathogen load, species and type, the life stage of tick, and duration of tick feeding influence the risk of pathogen transmission and the development of symptomatic infections. The present study focuses on the *Borrelia* content in those ticks with the aim to investigate if regions with different incidences of LB can be distinguished by studying the prevalence and diversity of *Borrelia* species in their respective tick populations. We expect that regions with high incidence of LB also have a high prevalence of *Borrelia* infected ticks, and vice versa, which has previously been demonstrated in other European regions [10,11]. We also wanted to investigate if there are any tick stage-related differences regarding prevalence and diversity of *Borrelia* species. Finally, we wanted to examine how the *Borrelia* load in ticks that have bitten humans is influenced by the duration of tick feeding. To investigate this, ticks that had bitten humans in two regions with different incidences of LB (located in the Finnish Åland Islands and Southernmost Sweden) and two regions with unknown incidences of LB (Northern Sweden and South Central Sweden) were collected. The duration of tick feeding was estimated by microscopic methods and *Borrelia* were detected and quantified by real-time PCR followed by species determination. The prevalence and diversity of *Borrelia* species found in the tick populations collected from the different regions were compared. We also compared the prevalence and diversity of *Borrelia* species in ticks at different life stages. In addition, the *Borrelia* load in ticks that had fed on humans was related to the duration of tick feeding.

## Materials and Methods

### Ethics Statement

Ethical permission for the TBD STING-study was granted by the Ethics Committee of the Medical Faculty, Linköping University (M132-06), and by the local Ethics Committee of the Åland Health Care, 2008-05-23.

### Selection of regions and collection of ticks

**Figure 1:**
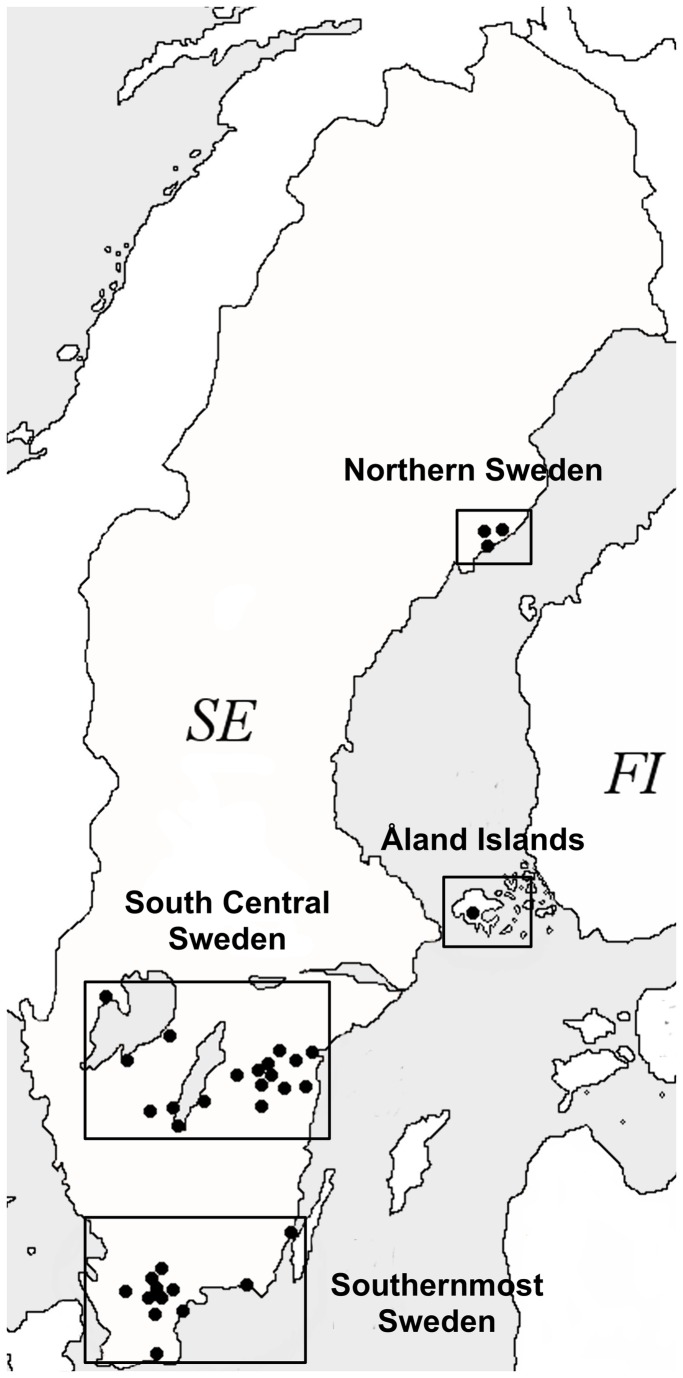
Map, showing the four regions where the 34 primary health care centers (PHCs) are located. Southernmost Sweden (10 PHCs), South Central Sweden (20 PHCs), Northern Sweden (3 PHCs), and Åland Islands (1 PHCs). SE: Sweden, FI: Finland.

Four regions: the Finnish Åland Islands and three separate regions of the Swedish mainland were selected, based on known or expected differences in LB incidences (Figure 1). The Åland Islands are a hyper-endemic region with an annual LB incidence in the range of 710-890/100,000 inhabitants [21], and a seroprevalence of 19.7% [22]. The region of Southernmost Sweden has an annual LB incidence in the range of 69-464/100,000 [23,24]. The LB incidence in South Central and Northern Sweden are unknown. In South Central Sweden, however, we previously reported a *Borrelia* seroprevalence of 11% among tick-bitten humans in the county of Östergötland [20], a lower seroprevalence than in the general population on the Åland Islands. 

Individuals aged 18 years or older were recruited into the TBD STING-study if they had recently been bitten by a tick. The study subjects were asked to bring their tick(s) to one of the 34 participating primary health care centers (PHCs) located in the four studied regions (Figure 1). At the initial visit at the PHCs, the bitten persons signed a written consent to participate, donated tick(s), blood sample, and completed a health questionnaire. Another blood sample was drawn and a second health questionnaire was filled in at a three-month follow-up visit to the PHC. Ticks and blood samples were transported to Linköping University within three days, where they were frozen at -70°C. Ticks were collected from May 2008 to November 2009. 

### Identification and measurement of ticks

Ticks were photographed and measured dorsally and ventrally, using a USB-microscope (Dino-Lite Long AM4013TL, AnMo Electronics Corp., Taiwan) to determine species, life stage, sex of adults, and to estimate feeding duration for adult female ticks and nymphs using coxal and scutal indices as described elsewhere [25]. 

### Tick homogenization, total nucleic acid extraction and reverse-transcription of nucleic acid

Forty-six ticks, one positive control (5 µl of *Borrelia burgdorferi* sensu stricto B31 ATCC 35210 [10^8^ cells/ml]), and one negative control, H_2_O, in separate 2 ml safe-lock microcentrifuge tubes were processed simultaneously. For each individual tick sample, 450 µl RLT buffer (Qiagen, Hilden, Germany) supplemented with 1% β-Mercaptoethanol (Sigma-Aldrich Sweden, Stockholm, Sweden) was added to each tube together with one 5-mm stainless steel bead (Qiagen). Ticks were homogenized for 2 min at 25 Hz at RT, using a TissueLyser (Qiagen). After a centrifugation step (3 min at 20,000 × g), 400 µl of the supernatants were collected in separate 2 ml micro tubes followed by automated total nucleic acid (NA) extraction. 

Total NA was extracted with a BioRobot M48 Workstation (Qiagen), using the MagAttract^®^ RNA Tissue Mini M48 Kit (Qiagen), according to the manufacturer's instructions with the exception of not adding DNase to the supplied RDD buffer, thus obtaining total NA including DNA. This will allow targeting non-coding regions (DNA) of the *Borrelia* genome as PCR-template for species determination. NA was eluted in a final volume of 50 µl RNAse free water. Twenty µl NA extract was used for reverse-transcribed NA (RTNA) synthesis, the remaining NA extract was stored at -70°C. 

RTNA synthesis was performed using illustra™ Ready-to-Go RT-PCR Beads kit (GE Healthcare, Amersham Place, UK) according to manufacturer's protocol, and 10 µl (0.25µg/µl) random hexamer primers (pd(N)_6_) were incubated with 20 µl NA for 5 min at 97°C. Beads were dissolved in 20 µl RNAse free water and transferred to the solution containing NA and primers, followed by incubation for 30 min at 42°C, and subsequently for 5 min at 95°C, resulting in a final volume of 50 µl RTNA. Pipetting was performed using a CAS-1200 pipetting robot (Corbett Robotics Inc., San Francisco, CA) and incubation using a PTC-100 thermal cycler (M. J. Research, Inc., Waltham, MA).

### LUX real-time PCR assay used for detection and quantification of *Borrelia*

Two microliters of RTNA per reaction were used in a Light Upon eXtension^TM^ (LUX) real-time PCR assay to detect and quantify *Borrelia* 16S rRNA, using genus-specific primers, as previously described [2]. PCR reagents and template RTNA were added to 96-well real-time PCR plates using CAS-1200 pipetting robot (Corbett Robotics Inc.).

To quantify the number of *Borrelia* cells, a serial dilution of plasmid standard was used. The plasmid contained the target sequence of the LUX real-time PCR assay, spanning the nucleotides 465284 - 465556 of the *B. burgdorferi* ss B31 chromosome (acc. no NC_001318.1). PCR product of the target sequence was synthesized and cloned into the pPCR-Script Amp SK(+) cloning vector (Stratagene, La Jolla, CA) according to the manufacturer's instructions.

### Estimation of real-time PCR inhibition

To estimate real-time PCR inhibition, a representative subset (15%) of all the extracted tick samples (61 adult ticks, 216 nymphs, 9 larvae, and 46 ticks that could neither be determined to species nor life stage (ND) was analyzed with a SYBR green real-time PCR assay to detect the *Ixodes* tick mitochondrial 16S rRNA, as previously described [2]. The *Ixodes* tick mitochondrial 16S rRNA was detected in all samples. The range of quantification cycle (Cq) values differed according to the life stage of the tick. The Cq values for adult ticks ranged from 9 to 20 (median 13, interquartile range [IQR] 11-15); for nymphs 9 to 21 (median 13, IQR 12-16), and for larvae 12 to 22 (median 16, IQR 13-21). Cq values for adult females and nymphs did not correlate with time of tick feeding. Cq values for the ND samples ranged from 10 to 37 (median 22, IQR 16-32). ND samples showed a higher median compared to the other groups, which was probably due to poor condition of the ticks. 

Possible PCR inhibition for the SYBR green real-time PCR assay was identified by measuring amplification performance using dilution series of samples to be targeted. Serial dilutions (1:1, 1:10, 1:100, and 1:1000) of RTNA processed from six fully engorged ticks (three adult ticks and three nymphs) were prepared in RNAse free water and analyzed, as described above. The amplification of undiluted and diluted samples showed parallel curves with a Cq difference of 3.4-3.5 with dilution. This was considered as PCR non-inhibitory. 

To check for possible inhibition in the *Borrelia* 16S rRNA LUX real-time PCR assay, serial dilutions (1:1, 1:10, 1:100, and 1:1000) of RTNA from three fully engorged adult ticks containing *B. valaisiana*, *B. burgdorferi* ss, and *B. spielmanii*, respectively, and three fully engorged nymphal ticks containing *B. afzelii*, *B. garinii*, and *B. miyamotoi*, respectively, were prepared in RNAse free water and analyzed, as described earlier. Prior to the test, the *Borrelia* species of these samples had been determined with the use of the 5S-23S and 16S-23S intergenic spacer PCR assays, as described below. The amplification of undiluted and diluted samples for all *Borrelia* species showed parallel curves with a highest Cq difference of 3.4 - 3.8 with dilution. Since the Cq values corresponds to an amplification efficiency between 83% and 97%, undiluted samples were used to screen RTNA samples for the presence of *Borrelia*.

### Comparison of LUX real-time PCR sensitivity targeting 16S rRNA (RTNA) and 16S rRNA gene (DNA)

We previously reported a LUX real-time PCR detection limit of fewer than 10^1^ *16S* rRNA gene copies of *B. burgdorferi* ss (B31) when DNA was used as template [2]. Ornstein and Barbour (2006) reported a 100-fold higher numbers of copies per *Borrelia* cell for 16S rRNA compared to *16S* rRNA gene (DNA) [26]. Their findings also indicated that rRNA copy numbers in *Borrelia* cells was less affected by the cell’s growth phase than DNA copy numbers, i.e. rRNA copies may be more appropriate to use to quantify *Borrelia* cells under different growth conditions. Here, we wanted to compare the LUX real-time PCR sensitivity targeting 16S rRNA (RTNA) and *16S* rRNA gene (DNA). *B. burgdorferi* ss (B31) were cultivated in BSK medium and counted in phase-contrast microscope, as previously described [2]. Two 10-fold serial dilutions of *B. burgdorferi* ss (B31) ranging from 10^7^ to 10^1^ were prepared in PBS and subsequently used for either total NA extraction followed by RTNA synthesis, as described above, or DNA extraction using DNeasy blood and tissue kit (Qiagen, Hilden, Germany), as previously described [2]. This was followed by LUX real-time PCR amplification, as described above. Figure S1 shows a comparison of Cq values obtained with the 16S rRNA (analyzed template RTNA) and *16S* rRNA gene (analyzed template DNA) tests. The mean Cq values were approximately 5 units (range 4.2 - 5.3) lower per dilution step with RTNA than DNA (data not shown), indicating a 10 to 100-fold higher number of 16S rRNA-molecules than *16S* rRNA genes per *Borrelia* cell, with the assumption of 100% yield in RTNA synthesis and equal yields of total NA and DNA extraction, respectively. The mean efficiency of the LUX real-time PCR for three replicates of each dilution step with RTNA and DNA was 91% (-3.58 ± 0.02, r^2^ = 0.99). 

### Conventional PCR assays and sequencing method used for *Borrelia* species identification

To determine the *Borrelia* species in LUX real-time PCR positive samples, a nested, conventional PCR assay using primers targeting the non-coding intergenic spacer region (IGS) between *5S* rRNA and *23S* rRNA genes was applied, as previously described [2]. Tick samples positive for *Borrelia* in the LUX real-time PCR that failed to produce PCR products with primers targeting the 5S-23S IGS were analyzed with primers targeting the 16S-23S IGS [27]. This allowed identification of other *Borrelia* species, such as *B. miyamotoi*, that lack the 5S-23S IGS.

Tick samples positive in the LUX real-time PCR that failed to produce PCR products with primers targeting both 5S-23S IGS and 16S-23S IGS were regarded as untypeable. 

Sequencing of all PCR products was performed by Macrogen Inc. (Seoul, South Korea). Mixed sequencing chromatograms were analyzed using the RipSeq Mixed web application (http://www.ripseq.com/login/login.aspx) (iSentio, Bergen, Norway). The RipSeq Mixed algorithm searches against the ‘5S-23S rRNA intergenic spacer’ database containing 43 sequences from six different *Borrelia* species. Non-mixed chromatograms were initially edited using BioEdit Software (V7.0) (Tom Hall, Ibis Therapeutics, Carlsbad, CA) followed by a standard BLAST search against the GenBank database (http://blast.ncbi.nlm.nih.gov). To confirm the genetic identity of the *Borrelia* species detected in ticks, 5S-23S and 16S-23S sequences were aligned with the corresponding *B. afzelii*, *B. garinii*, *B. valaisiana*, *B. burgdorferi* ss, *B. miyamotoi*, *B. spielmanii*, and *B. lusitaniae*, sequences available in GenBank using ClustalW2 [28]. Within each species, sequences of the intergenic spacer regions showed a sequence similarity between 94 and 100%. 

5S-23S IGS and 16S-23S IGS obtained in this investigation have been deposited in GenBank with accession numbers JX909617 to JX910061 (n = 445), and JX910062 to JX910079 (n = 18), respectively. 

### Statistical analysis

Fisher’s exact test or Chi square test were applied to compare the prevalence of *Borrelia* infected ticks and number of different *Borrelia* species between the regions, and within the collecting season (comparison between months). Comparisons of the *Borrelia* load between different *Borrelia* species were analyzed using Kruskal-Wallis test, which, if significant, was followed by Dunn´s post test. The Spearman correlation coefficient was used to investigate any correlation between the duration of tick feeding and the *Borrelia* load in ticks. The test was applied on ticks in different life stages. Mann-Whitney test was used to compare the loads of *Borrelia* in ticks that had fed for more than 36 hours with ticks that had fed for less than 36 hours. Results were reported as median values with interquartile range. Statistical analyses were performed and graphs were drawn using GraphPad Prism version 5.00 for Windows (GraphPad Software, San Diego, CA). p-values ≤ 0.05 were considered significant.

## Results

### Ticks collected

A total of 2,154 ticks, including 15 adult males (1%), 496 adult females (23%), 1,510 nymphs (70%), and 87 larvae (4%) were collected (Table 1). Forty-six (2%) ticks were so damaged that neither life stage nor species could be determined. The remaining ticks were morphologically determined as *I. ricinus*. *Ixodes* mitochondrial 16S rRNA was detected by PCR in all damaged ticks; these may be assumed to be *I. ricinus*, as this is the only *Ixodes* tick known to bite humans in Sweden.

**Table 1:** Borrelia prevalence and species in ticks, collected from tick-bitten individuals in different regions

Region**^*a*^**	No. (%) of ticks examined**^*b*^**	No.(%) of positive ticks**^*c*^**	No. (%) of ticks containing *Borrelia* species determined by nucleotide sequencing**^*d*^**								
			*B. a*	*B. g*	*B. v*	*B. b*	*B. m*	*B. s*	*B. l*	Mixed	UT
Total	2154 (100)	556 (26)	279 (50)	109 (19)	35 (7)	21 (4)	11 (2)	6 (1)	2 (1)	2 (1)	91 (15)
Adult male	15 (1)	5 (33)	2 (40)	1 (20)	1 (20)	0	0	0	0	0	1(20)
Adult female	496 (23)	175 (36)	68 (38)	44 (25)	16 (9)	11 (6)	5 (3)	1 (1)	1 (1)	1 (1)	28 (16)
Nymph	1510 (70)	369 (25)	208 (56)	62 (17)	18 (5)	9 (2)	6 (2)	5 (1)	1 (1)	1 (1)	59 (15)
Larva	87 (4)	0	0	0	0	0	0	0	0	0	0
ND**^*e*^**	46 (2)	7 (15)	1 (14)	2 (29)	0	1 (14)	0	0	0	0	3 (43)
A	541 (25)	133 (25)	68 (51)	24 (18)	8 (6)	7 (5)	1 (1)	0	1 (1)	1 (1)	23 (17)
Adult male	1 (1)	0	0	0	0	0	0	0	0	0	0
Adult female	115 (21)	41 (36)	16 (39)	11 (27)	4 (10)	3 (7)	0	0	0	1 (2)	6 (15)
Nymph	411 (76)	92 (23)	52 (57)	13 (14)	4 (4)	4 (4)	1 (1)	0	1 (1)	0	17 (19)
Larva	14 (2)	0	0	0	0	0	0	0	0	0	0
B	771 (37)	236 (31)	123 (52)	41 (17)	12 (5)	9 (4)	8 (3)	1 (1)	1 (1)	0	41 (17)
Adult male	9 (1)	5 (56)	2 (40)	1 (20)	1 (20)	0	0	0	0	0	1 (20)
Adult female	254 (33)	99 (39)	42 (42)	22 (22)	7 (8)	5 (5)	4 (4)	0	1 (1)	0	18 (18)
Nymph	503 (65)	132 (26)	79 (60)	18 (13)	4 (3)	4 (3)	4 (3)	1 (1)	0	0	22 (17)
Larva	5 (1)	0	0	0	0	0	0	0	0	0	0
C	19 (1)	2 (11)	1 (50)	0	1 (50)	0	0	0	0	0	0
Adult male	0	0	0	0	0	0	0	0	0	0	0
Adult female	9 (47)	0	0	0	0	0	0	0	0	0	0
Nymph	9 (47)	2 (22)	1 (50)	0	1 (50)	0	0	0	0	0	0
Larva	1 (6)	0	0	0	0	0	0	0	0	0	0
D	777 (37)	178 (23)	86 (48)	42 (24)	14 (8)	4 (2)	2 (1)	5 (3)	0	1(1)	24 (13)
Adult male	5 (1)	0	0	0	0	0	0	0	0	0	0
Adult female	118 (15)	35 (30)	10 (28)	11 (30)	5 (14)	3 (8)	1 (3)	1 (3)	0	0	4 (11)
Nymph	587 (74)	143 (24)	76 (53)	31 (21)	9 (6)	1 (1)	1 (1)	4 (3)	0	1 (1)	20 (14)
Larva	67 (8)	0	0	0	0	0	0	0	0	0	0

*a* Regions: A, Southernmost Sweden; B, South Central Sweden; C, Northern Sweden; D, Åland Islands

*b* Percent and numbers in shaded rows indicate proportion of the total number of ticks examined, all other percent and numbers indicate proportion within respective region

*c* Number of ticks yielding a positive outcome by real-time PCR analysis

*d* Abbreviations: *B. a*, *B. afzelii*; *B. g*, *B. garinii*; *B. v*, *B. valaisiana*; *B. b*, *B. burgdorferi* sensu stricto; *B. m*, *B. miyamotoi*; *B. s*, *B. spielmanii*; *B. l*, *B. lusitaniae*; UT, untypeable

*e* ND, developmental stage could not be determined due to damages. ND-ticks are only presented in the column showing total collected ticks

Tick life stage distribution differed between the study regions (Table 1). A higher proportion of adult ticks were collected in Sweden (21%; Southernmost Sweden, 33%; South Central Sweden) than in the Åland Islands (15%, p < 0.001, respectively). Seventy-seven percent of larvae (67 of 87) were collected from the Åland Islands. Due to the low number of ticks collected from Northern Sweden (n = 19), these ticks were excluded from further statistical analysis.

### Every fourth tick contained *Borrelia*

Overall, 26% of the collected ticks (556 of 2,154) contained *Borrelia* (Table 1). Adult female ticks had a higher *Borrelia* prevalence (36%) compared to nymphs (25%, p < 0.001). *Borrelia* was not detected in any of the 87 larvae.

The *Borrelia* prevalence in ticks collected from South Central Sweden (31%) was higher compared to Southernmost Sweden (25%, p < 0.05), and to Åland Islands (23%, p < 0.01). Only 11% (2 of 19) of the ticks collected from Northern Sweden contained *Borrelia*. The *Borrelia* prevalence in adult ticks collected from South Central Sweden was higher (40%, 104 of 263) compared to adult ticks collected from Åland Islands (28%, 35 of 123, p < 0.05). No other significant differences in the *Borrelia* prevalence in adult ticks between the regions were noticed. The *Borrelia* prevalence in nymphal ticks collected from the four regions varied between 22% and 26%, but did not differ significantly (Table 1). No significant seasonal variations (24% - 29%) in the prevalence of *Borrelia* infection in ticks were noticed (data not shown).

### High diversity of *Borrelia* species found in the ticks

Seven different *Borrelia* species were identified by sequence analysis of the 5S-23S and 16S-23S IGS (Table 1). *B. afzelii* was the predominant species and was detected in 50% of all ticks containing *Borrelia*, followed by *B. garinii* (19%), *B. valaisiana* (7%), *B. burgdorferi* ss (4%), *B. miyamotoi* (2%), *B. spielmanii* (1%), *B. lusitaniae* (1%), mixed infection of *Borrelia* species (1%), and 15% were untypeable. A similar descending order in distribution of *Borrelia* species was observed in all regions, except in Northern Sweden where only two (of 19) ticks were *Borrelia* infected. Among the ticks with mixed infection of *Borrelia* species (n = 2), an adult female tick contained *B. garinii* and *B. valaisiana* and a nymphal tick contained two strains of *B. burgdorferi* ss. 

**Figure 2:**
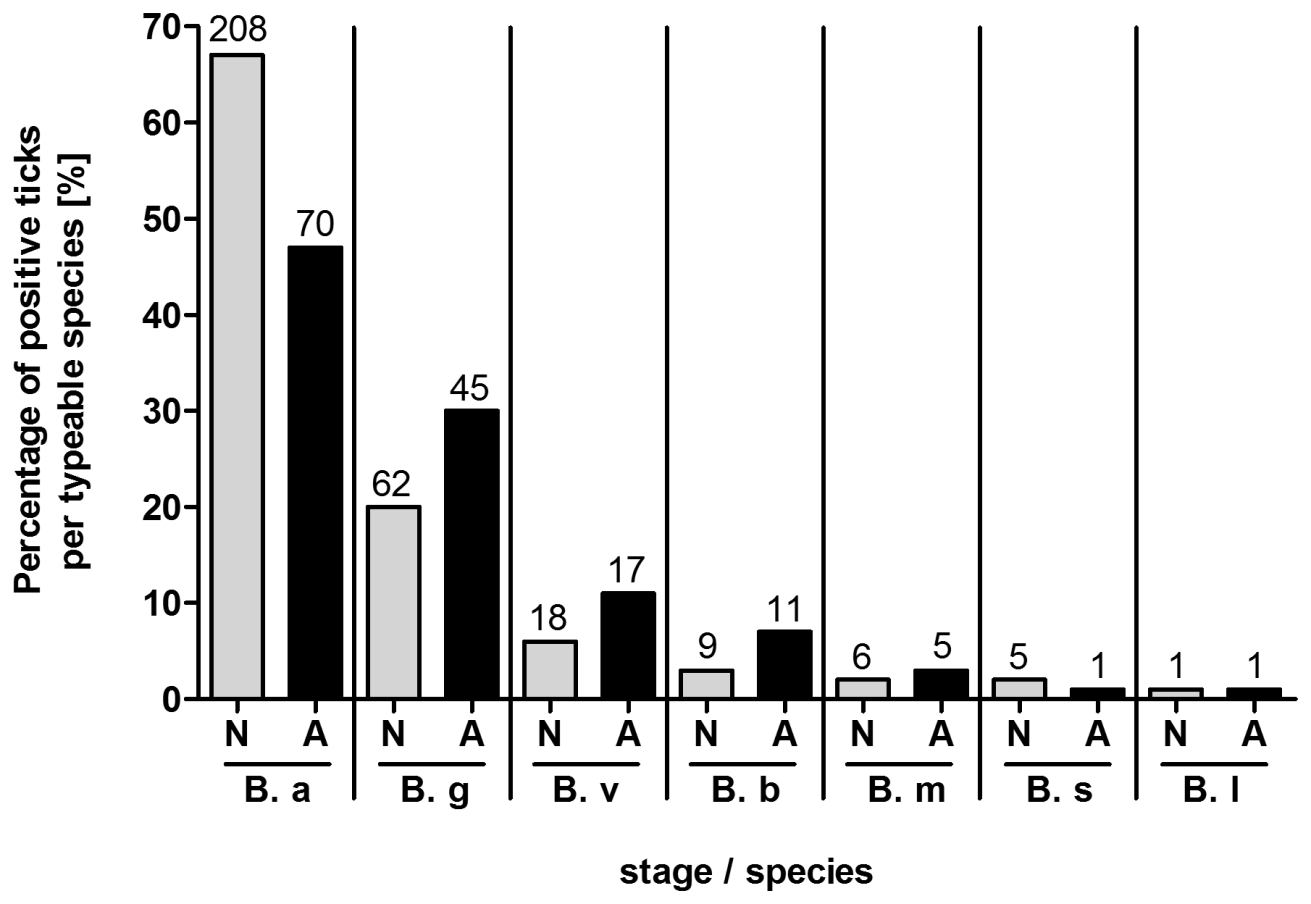
Distribution of *Borrelia* species in nymphs (N) and adults (A). The percentage of positive ticks per typeable species is given. The numbers of infected ticks examined are indicated above the bars. Abbreviations: *B*. *a*, *B. afzelii*; *B*. *g*, *B. garinii*; *B*. *v*, *B. valaisiana*; *B*. *b*, *B. burgdorferi* sensu stricto; *B*. *m*, *B. miyamotoi*; *B*. *s*, *B. spielmanii*; *B*. *l*, *B. lusitaniae*.

Of all samples that were determined to *Borrelia* species, *B. afzelii* was more prevalent in nymphs (67%, 208 of 309) than in adult ticks (46%, 70 of 150, p < 0.001) (Figure 2). *B. garinii* and *B. valaisiana* showed the opposite pattern and were more prevalent in adult ticks (30%, 45 of 150, and 11%, 17 of 150, respectively) than in nymphs (20%, 62 of 309, and 6%, 18 of 309, respectively) (p < 0.05). No significant regional or seasonal differences in prevalence of *Borrelia* species were detected.

### The *Borrelia* load in the ticks differed according to *Borrelia* species

**Figure 3:**
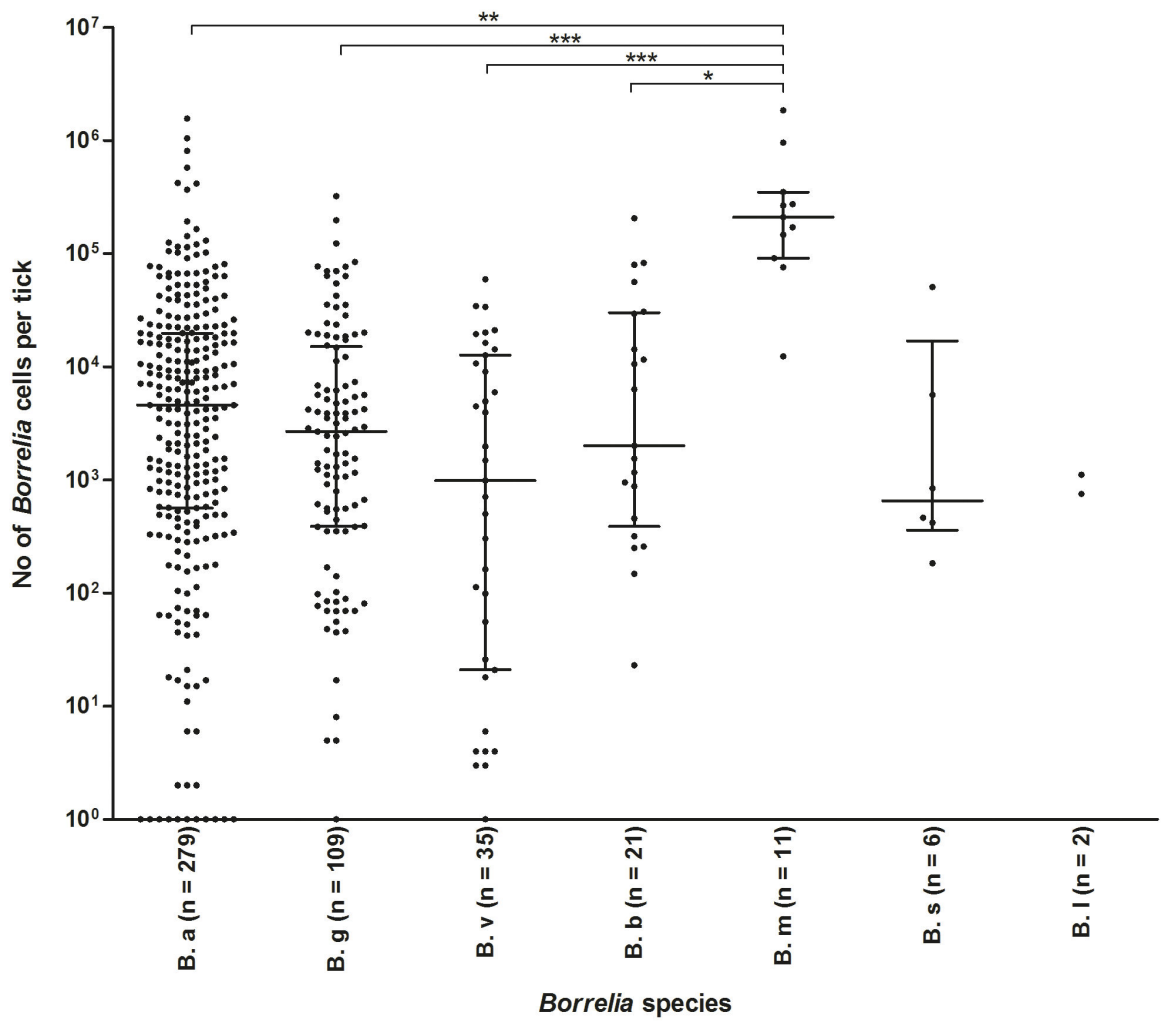
*Borrelia* species plotted against the number of *Borrelia* cells per tick. Horizontal lines indicate the median, with upper and lower quartiles. *, p < 0.05; **, p < 0.01; ***, p < 0.001. Abbreviations: *B*. *a*, *B. afzelii*; *B*. *g*, *B. garinii*; *B*. *v*, *B. valaisiana*; *B*. *b*, *B. burgdorferi* sensu stricto; *B*. *m*, *B. miyamotoi*; *B*. *s*, *B. spielmanii*; *B*. *l*, *B. lusitaniae*.

According to the LUX real-time PCR assay, the *Borrelia* load per tick ranged from 1.0 × 10° to 1.8 × 10^6^ cells per tick (Figure 3) with a median of 1.6 × 10^3^. No significant difference in *Borrelia* load between adult ticks and nymphs was observed (medians 1.5 × 10^3^, and 1.7 × 10^3^, respectively) (data not shown). This was also the case for adults and nymphs with estimated feeding times less than 24 hours (medians 6.3× 10^3^, and 3.2 × 10^3^, respectively) (data not shown). 

Ticks infected with *B. miyamotoi* had a significantly higher *Borrelia* load (median of 2.1 × 10^5^ cells per tick) compared to ticks infected with *B. afzelii* (median 4.5 × 10^3^, p < 0.01)*, B. garinii* (median 2.7 × 10^3^, p < 0.001)*, B. valaisiana* (median 1.0 × 10^3^, p < 0.001), and *B. burgdorferi* ss (median 2.0 × 10^3^, p < 0.05) (Figure 3). No other significant differences in *Borrelia* load between species were noticed. Ticks infected with untypeable *Borrelia*, had a significantly lower *Borrelia* load (median of 1.2 × 10^1^ cells per tick) compared to ticks infected with typeable *Borrelia* species (p < 0.001) (data not shown). The *Borrelia* load in the ticks with mixed infection of *Borrelia* species contained 6.6 × 10^2^ and 6.0 × 10^1^ cells per tick, respectively.

### The *Borrelia* load in adult female ticks differed according to estimated time of tick feeding

**Figure 4:**
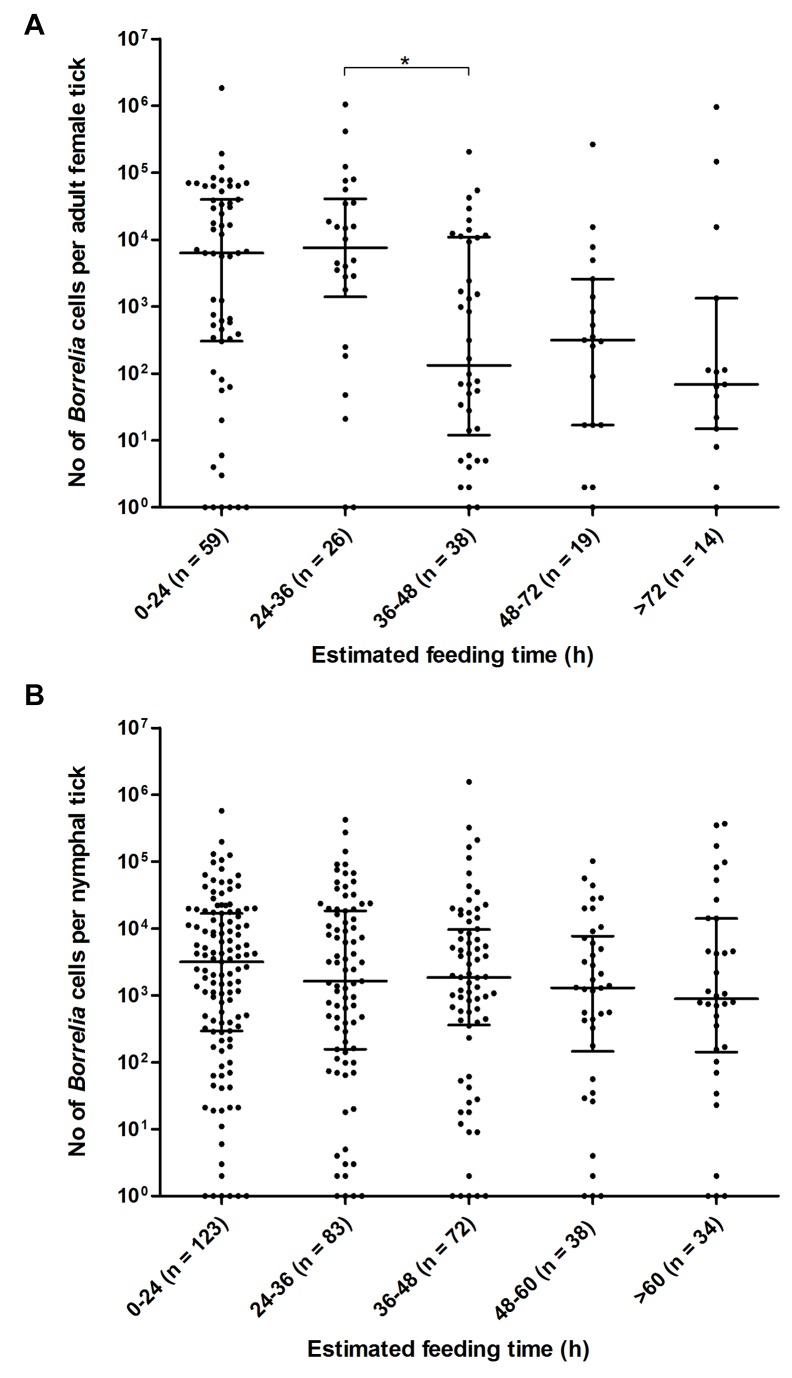
Tick feeding-time (hours) plotted against the number of *Borrelia* cells in A. adult female ticks, and B. nymphal ticks. Horizontal lines indicate the median, with upper and lower quartiles. *, p < 0.05.

**Figure 5:**
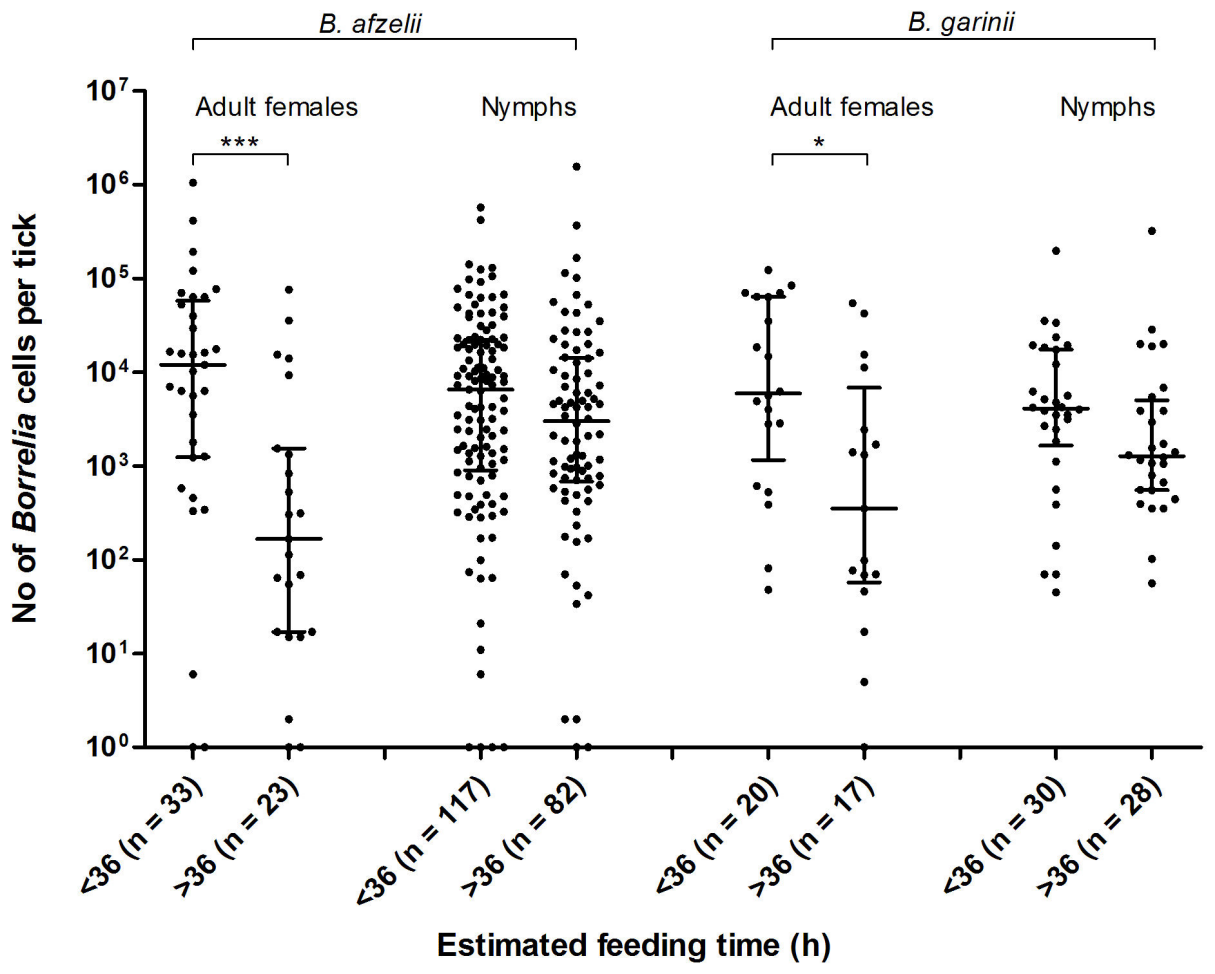
Tick feeding-time (hours) plotted against the number of *Borrelia* cells in ticks of different stages. Adult female ticks and nymphs infected with *B. afzelii* or *B. garinii*. Horizontal lines indicate the median, with upper and lower quartiles. *, p < 0.05; ***, p < 0.001.

When the *Borrelia* load, quantified in adult females (n = 156), and in nymphs (n = 350), was plotted against their respective feeding-time (hours), a significant correlation between the *Borrelia* load in adult females with estimated time of tick feeding was found (Spearman correlation = -0.25, p < 0.01). When the estimated time of tick feeding was stratified into intervals, we noticed a significantly lower *Borrelia* load in the adult females after 36 hours of feeding (Figure 4A). This was the case for both *B. afzelii* and *B. garinii* (Figure 5). For the remaining *Borrelia* species numbers were too small to allow analysis. No significant correlation between the *Borrelia* load and time of nymphal tick-feeding was observed (Figure 4B). 

## Discussion

This study detected that 26% of the ticks that had bitten humans in four regions of Sweden and Åland Islands were *Borrelia* infected. This is considerably higher than the overall mean prevalence of *Borrelia* infection in ticks in Europe (13.6%) [10]. In agreement with previous reports [10], adult ticks were more often infected than nymphs, which is probably related to the higher number of blood meals ingested by the adult ticks. None of the larvae was infected with *Borrelia*, which is in agreement with previous results indicating that species belonging to the *B. burgdorferi* sl complex lack a trans-ovarial transmission route [29]. 

The regions of Åland Islands and Southernmost Sweden are estimated to have different incidences of LB. However, the tick populations collected from these regions exhibited only minor differences in the prevalence of *Borrelia*; therefore, it appears insufficient to use the *Borrelia* prevalence in a tick population as the only indicator to reflect the incidence of LB among humans. This indicates that other factors such as tick density, human behavior, exposure to ticks and frequency of tick bites may influence the incidence of LB in a region. Alternatively, since LB is not a mandatory notifiable disease in these regions, we have to rely on estimates of the LB incidences, which may be fraught with some uncertainty; it is possible that there could be a similar incidence of LB in both Åland Islands and Southernmost Sweden. Interestingly, the prevalence of *Borrelia* infected adult ticks collected from South Central Sweden was significantly higher (40%) compared to the prevalence found in adult ticks collected from the Åland Islands (28%). A high prevalence of *Borrelia* in a tick population could be due to a long season of tick activity and a low mortality rate for ticks, as well as a high access to *Borrelia* reservoir hosts [30]. However, the reason for the high prevalence of *Borrelia* infected ticks in South Central Sweden is unknown and needs to be further investigated.

Seven *Borrelia* species were detected. *B. afzelii* was the dominating species, which has frequently been associated to have rodent populations as reservoir hosts [31,32]. Thereafter followed *B. garinii* and *B. valaisiana*; two species that have been linked to avian populations [31]. Interestingly, *B. afzelii* was significantly more prevalent in nymphs than in adult ticks, while *B. garinii* and *B. valaisiana* showed the opposite pattern, indicating different host preferences of ticks according to their life stages. Nymphs are developed from larvae, which frequently feed on small rodents [33], and adult ticks are developed from nymphs, which frequently feed on birds [34]. *B. burgdorferi* ss, a species that has the ability to persist in a wide range of vertebrates [9], was also detected. However, to estimate the absolute contribution of a reservoir host to the prevalence of *Borrelia* infection in a tick population, one must first accurately identify the host, which could be achieved by analyzing the ticks’ previous blood-meal [35]. In the present study, *B. miyamotoi*, *B. lusitaniae*, and, for the first time in ticks collected from Sweden and Åland Islands, *B. spielmanii* were all detected in a low number of ticks and were geographically restricted. There could be many reasons for this, e.g. these species might have a narrow spectrum of reservoir hosts in the studied areas or they might have lower transmission efficiency from reservoir hosts to ticks. Only 1% of all ticks contained a mixed *Borrelia* infection, which corroborates previous reports from the Nordic countries Sweden and Norway [2,36], but is lower than the average percentage (13%) reported in a meta-analysis of European studies [10]. The reason for the lower occurrence of mixed *Borrelia* infection in ticks from the Nordic countries is unknown, but it may be due to elimination of existing *Borrelia* infections during tick feeding [8,9]. We also detected *Borrelia* that could not be determined to species with the use of the IGS sequences, probably due to the low number of *Borrelia* cells found in those ticks.

Even though no significant differences in prevalence of *Borrelia* species between the different regions were noticed, higher proportions of *B. garinii* infected adult ticks and nymphs were found in Åland Islands, compared to the regions in Sweden (Table 1). Further, 80% (5 of 6) of the ticks infected with *B. spielmanii* were collected from the Åland Islands. Notably, 70% (8 of 11) of the ticks infected with the RF-causing *B. miyamotoi* were collected from the region in South Central Sweden (Table 1), where clinicians should be advised to be observant for the signs and symptoms of RF among patients. In Northern Sweden, the prevalence of *Borrelia* in ticks was low (11%). However, this figure is uncertain since few ticks (n = 19) were collected from this region. The low number of ticks collected probably reflect the low abundance of the ticks in this region [37].

The number of *Borrelia* cells in the infected ticks ranged from fewer than ten to more than a million. The ticks infected with *B. miyamotoi* had a significantly higher *Borrelia* load per tick compared to the ticks infected with other *Borrelia* species (Figure 3). The reason for this is unknown but the ticks infected with *B. miyamotoi* could have obtained a higher load of this species from their previous blood meal; reservoir hosts for *B. miyamotoi* have shown to contain a higher load of *B. miyamotoi* in their blood compared to other *Borrelia* species [38]. Another explanation for the high load of *Borrelia* cells found in the *B. miyamotoi* infected ticks could be that this species has a high survival rate during the tick molting process. However, this has never been investigated. Nevertheless, a high load of *Borrelia* in a feeding tick could facilitate a transmission of *Borrelia* spirochetes from tick to host [19].

We examined how the *Borrelia* load was influenced by the duration of tick feeding. After 36 hours of feeding, adult female ticks infected with *B. afzelii* and *B. garinii* contained a significantly lower *Borrelia* load compared to ticks with a shorter duration of feeding than 36 hours. The reason for this is unknown, but one could speculate that the lower *Borrelia* loads, observed in adult female ticks after 36 hours of feeding, reflect a transmission of these *Borrelia* species from tick to human. De Silva et al. (1995) observed that *B. burgdorferi* ss in *Ixodes* nymphs migrated from the gut to the salivary glands of the tick approximately 36 hours after they started to feed on mice [39]. However, to conclude that the present observation was a process of transmission, one would have to investigate the number of *Borrelia* cells an infected tick transmit when feeding on humans. When we first observed these differences in the *Borrelia* load, we questioned whether the lower *Borrelia* loads quantified in the adult female ticks that had fed for more than 36 hours represented PCR inhibition due to the greater volume of blood present. However, when evaluating the real-time PCR assay on fully engorged ticks spiked with *Borrelia* bacteria, no signs of inhibition were noticed. On the other hand, three days may have elapsed between sampling and freezing of ticks at -70 °C. This delay may have led to a higher degradation of *Borrelia* bacteria in ticks that had ingested a greater volume of blood, thus a higher level of innate immune cells and elements of the complement cascade. Compared to adult female ticks, nymphs ingest a smaller volume of blood. In addition, we did not observe a significant reduction of the *Borrelia* load in nymphs that had fed for more than 36 hours. 

This study, which was conducted during two subsequent seasons, represents a “snapshot” of the prevalence and the diversity of *Borrelia* species in ticks that have fed on humans. However, to detect data trends in temporal and spatial shifts regarding these parameters, long-term studies are needed. A longitudinal study can also reveal the effect of weather and climate on the distribution of LB. Further, to predict and prevent human risk of exposure to LB, it is important to identify the *Borrelia* reservoir hosts and their abundance and distribution. It is also important to further investigate what impact the *Borrelia* load in a feeding tick might have on the probability of infection in humans. This could be achieved by relating the *Borrelia* load in the ticks to the serological and clinical response of the bitten persons. 

## Supporting Information

Figure S1 **Comparison of quantification cycle (Cq) values for 16S rRNA (RTNA) and 16S rRNA gene (DNA) using LUX real-time PCR assay.** Numbers of spiked *Borrelia* cells before total NA and DNA extraction are specified in the plot.
(TIF)
