# Supplementary figures and images for: Prevalence, Diversity, and Load of Borrelia species in Ticks That Have Fed on Humans in Regions of Sweden and Åland Islands, Finland with Different Lyme Borreliosis Incidences
Source: PLoS One. 2013 Nov 21;8(11):e81433. doi: 10.1371/journal.pone.0081433 (PMC3836827; doi:10.1371/journal.pone.0081433)

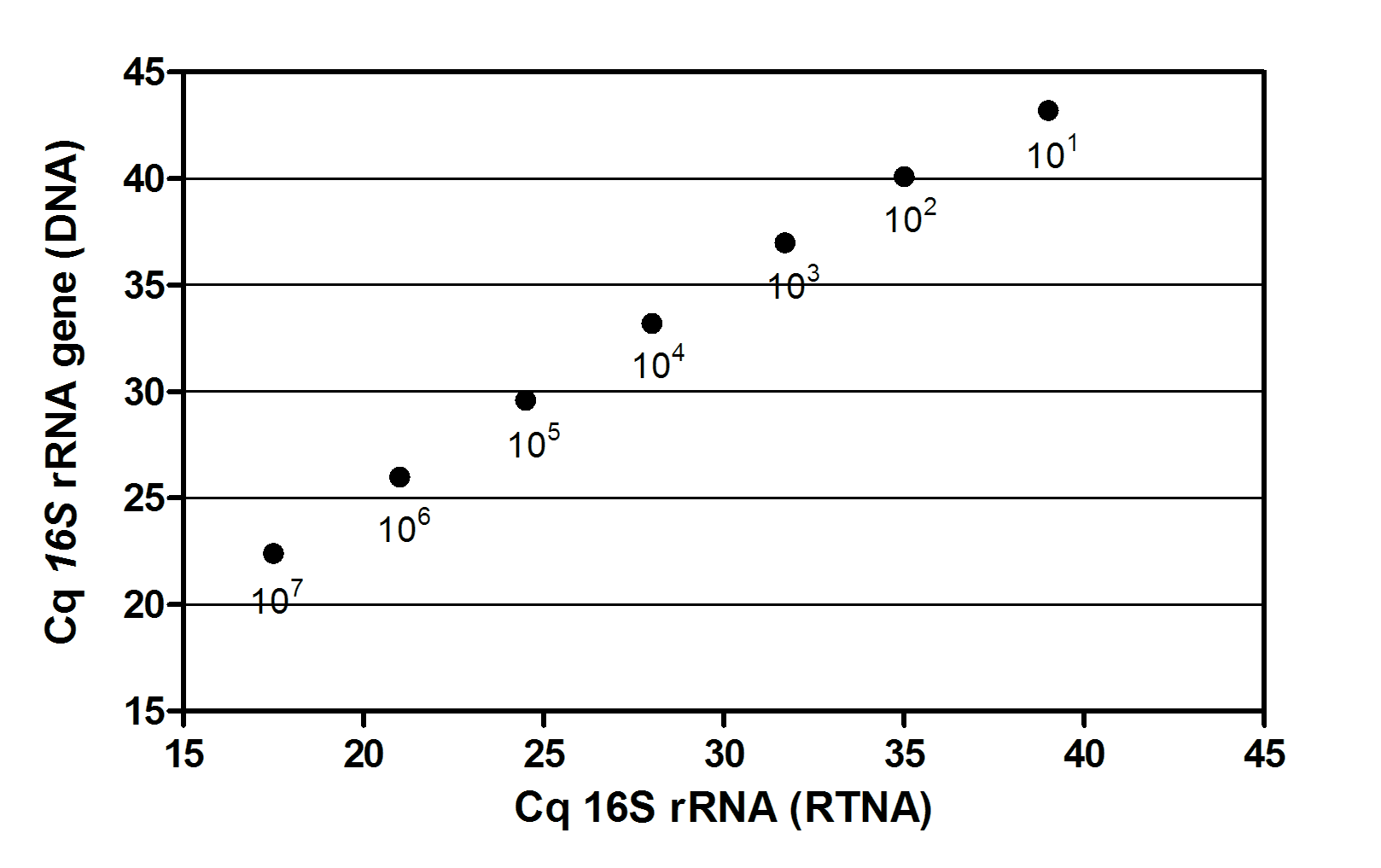

Supplement: Figure S1 — Comparison of quantification cycle (Cq) values for 16S rRNA (RTNA) and 16S rRNA gene (DNA) using LUX real-time PCR assay. Numbers of spiked Borrelia cells before total NA and DNA extraction are specified in the plot. (TIF) [file pone.0081433.s001.tif]
